# TMEM100 Modulates TGF-*β* Signaling Pathway to Inhibit Colorectal Cancer Progression

**DOI:** 10.1155/2021/5552324

**Published:** 2021-08-11

**Authors:** Huixia Li, Chuan Cheng, Weibo You, Jiujian Zheng, Jie Xu, Peng Gao, Jianping Wang

**Affiliations:** Department of Colorectal and Anal Surgery, Lishui Municipal Central Hospital, Affiliated Lishui Hospital of Zhejiang University, Lishui 323000, China

## Abstract

**Objectives:**

This study investigated the functional mechanism of transmembrane protein 100 (TMEM100) as a tumor inhibitor gene in CRC cells and offered a reference for the treatment of CRC.

**Methods:**

The mRNA expression data of CRC were acquired from the TCGA database to mine differentially expressed mRNAs. The role of TMEM100 in the progression of CRC cells was evaluated by MTT, colony formation, scratch healing, and Transwell assays. The influence of TMEM100 on the TGF-*β* signaling pathway was detected by western blot.

**Results:**

TMEM100 was markedly lowly expressed in CRC. CRC cell growth was significantly suppressed by overexpressing TMEM100 but noticeably facilitated by silencing TMEM100. Overexpression of TMEM100 inhibited the activation of the TGF-*β* signaling pathway, thus inhibiting malignant progression of CRC.

**Conclusion:**

TMEM100 is lowly expressed in CRC, which can suppress CRC cell growth by regulating the TGF-*β* signaling pathway.

## 1. Introduction

Colorectal cancer (CRC) ranks 3^rd^ concerning incidence rate (10.2%) and 2^nd^ regarding mortality rate (9.2%), which has become a serious threat to people's health [1]. Research has suggested that patients with early CRC tend to have better therapeutic effect [2]. However, most CRC sufferers are already at an advanced stage when being confirmed. There is still a lack of effective therapeutic method for advanced CRC sufferers, and over 90% of them have local recurrence and distant metastasis [3–5]. Accordingly, it is rather important to fully understand the exact molecular mechanism of occurrence and development of CRC and find the key regulatory gene in CRC in order to provide further guidance for CRC treatment.

Currently, studies have indicated that the mutation of many genes is vital in the development of CRC. For instance, the tumor suppressor genes P53 [6] and PTEN [7] tend to mutate and foster tumorigenesis in CRC. Besides, research has also showed that miRNA is able to repress the occurrence and metastasis of CRC by silencing K-ras [8]. A transmembrane protein (TMEM) acts as a channel to allow the movement of particular substances cross the biological membranes [9]. TMEM proteins perform important physiological functions in various cell types. For example, TMEM45A is associated with keratinization [10]; TMEM16 is closely related to cell autophagy [11]. In addition, TMEM proteins are reported to express differentially in various cancers and capable of regulating growth of tumors. For instance, TMEM48 is expressed notably high in non-small-cell lung cancer (NSCLC), while silencing it could significantly stimulate cell apoptosis and inhibit the adhesion, migration, invasion, and tumorigenesis of cells in nude mice [12]. TMEM100 is noticeably downregulated in gastric cancer (GC) tissue. Overexpressing TMEM100 hampers invasion and migration of GC cells but does not affect their growth. And TMEM100 expression could restore the chemosensitivity of GC cells to drugs such as 5 fluorouracil (5-Fu) and cisplatin [13]. Nevertheless, the role of TMEM100 in CRC has not been investigated so far.

Consequently, the present study explored the molecular mechanism by which TMEM100 regulated CRC cell growth via a set of *in vitro* experiments, so as to better understand CRC pathogenesis and provide a novel idea for clinical treatment of CRC.

## 2. Materials and Methods

### 2.1. Cell Incubation and Collection of Samples

Normal human colorectal cell line FHC and CRC cell lines HCT116 (ATCC® CCL-247EMT), HCT-15 (ATCC® CCL-225), NCI-H498 (ATCC® CCL-254), and SNU-C2B (ATCC® CCL-250) were purchased from Genetimes ExCell Technology, Inc. (Shanghai, China) and incubated in RPMI 1640 (Gibco, 11875093) medium added with 10% fetal bovine serum (FBS; Gibco, 10099141C). The incubation conditions were 37°C and 5% CO_2_.

Twenty pairs of CRC tumor and normal adjacent tissue samples of patients from the Department of Colorectal and Anal Surgery, Lishui Municipal Central Hospital, were collected with informed consent from all of them. After surgical resection, samples were immediately preserved in liquid nitrogen at -80°C for experiment. This experiment obtained the approval of the Ethics Committee of Lishui Municipal Central Hospital.

### 2.2. Gene Overexpression and Knockdown

Small interfering RNA (siRNA) targeting TMEM100 was designed by Sangon Biotech (Shanghai) Co., Ltd. pEGFP1 vector was applied to construct pEGFP1-TMEM100 and pEGFP1-Smad2/Smad3 recombinant plasmids. CRC cells were subjected to transfection with siRNA, recombinant plasmids, and corresponding controls by using Lipofectamine®3000 reagent (Invitrogen, USA).

### 2.3. Real-Time Quantitative PCR (qRT-PCR)

Total RNA was obtained from CRC tissue and cells by using TRIzol reagent (Invitrogen). Then, complementary DNA (cDNA) was obtained from extracted RNA with the Reverse Transcription Assay Kit (Invitrogen). The ABI 7900HT (Applied Biosystems, USA) was used to perform qRT-PCR with the miScript SYBR Green PCR Kit (Qiagen, Germany) under the following thermocycling conditions: initial denaturation at 95°C for 10 min, 95°C for 2 min, 40 cycles at 95°C for 5 s, and 60°C for 30 s. GAPDH served as an internal control for TMEM100. Quantitative value was expressed in the 2^-*ΔΔ*Ct^ value to analyze difference in the relative expression of TMEM100 between the control group and test group. The experiment was conducted in triplicate. Supplementary Table 1 listed the information of primers.

### 2.4. MTT Assay

NCI-H498 cells (5 × 10^3^) were placed into a 96-well plate. Each treatment consisted of three triplicates. At 1 d, 2 d, 3 d, 4 d, and 5 d, cells were added with sterile MTT solution (Beyotime) for proliferation evaluation as per the manufacturer's specification. At last, absorbance at 570 nm was identified by the microplate reader (Molecular Devices, Sunnyvale, CA, USA).

### 2.5. Colony Formation Assay

Transfected NCI-H498 cells (5 × 10^2^ cells/well) were cultured in a 6-well plate for 2 weeks. Thereafter, cells were treated with 4% paraformaldehyde for 15 min and dyed with 1 ml of 0.1% crystal violet for 30 min. Photos were taken, and colonies qualified were counted.

### 2.6. Scratch Healing Assay

NCI-H498 cells (1 × 10^6^) were placed into a 6-well plate. When 80% of cell confluency was achieved, a scratch was created on cells using the tip of a 200 *μ*l pipette. The floating cells were removed by washing the medium twice, after which cells were cultured for another 24 h in a fresh medium. The cells were observed and photographed using a microscope at 0 and 24 h.

### 2.7. Transwell Assay

About 2 × 10^4^ cells were inoculated into the Matrigel-coated upper compartment. The lower compartment was added with Dulbecco's modified Eagle's medium (DMEM) plus 10% FBS (Thermo Fisher, USA). After cells were incubated at 37°C for 48 h, noninvasive cells in the upper compartment were removed. While invasive cells in the lower compartment were stained with 0.5% crystal violet. Afterwards, cells were observed, photographed, and counted under a microscope.

### 2.8. Western Blot

After 48 h of transfection, cells were washed with precooled phosphate-buffered saline (PBS; Thermo Fisher, USA) in triplicate and then subjected to 10 min of lysis on ice by using whole-cell lysate. Proteins obtained were quantitated by applying BCA Protein Assay Kit (Thermo Fisher, USA), boiled along with 10 *μ*l loading buffer at 95°C for 10 min, and sequentially separated by sodium dodecyl sulfate-polyacrylamide gel electrophoresis (SDS-PAGE) at a voltage of 100 V. Then, the samples were loaded to nitrocellulose membranes at 100 mA within 120 min. Membranes were subsequently blocked in 5% BSA/TBST for further 60 min. Afterwards, primary rabbit antibodies were cultured with the membranes overnight at 4°C. Subsequently, the incubation of horseradish peroxidase- (HRP-) conjugated secondary antibody goat anti-rabbit IgG and the membranes was performed at room temperature for 120 min. Finally, enhanced chemiluminescence (ECL) assay kit (Solarbio, Beijing, China) was used to visualize protein bands and images were captured for further observation. Supplementary Table 2 exhibited details of antibodies. The experiment was conducted in triplicate.

### 2.9. Statistical Analysis

All data analyses were processed using SPSS 22.0 software. All measurement data were exhibited as mean ± standard deviation (SD). Student's *t*-test was used for analyzing differences between the two groups. ^∗^*p* < 0.05 indicated statistically significant difference, and ^∗∗^*p* < 0.01 indicated highly statistically significant difference.

## 3. Results

### 3.1. TMEM100 Shows Low Expression in CRC Tissue and Cells

**Figure 1:**
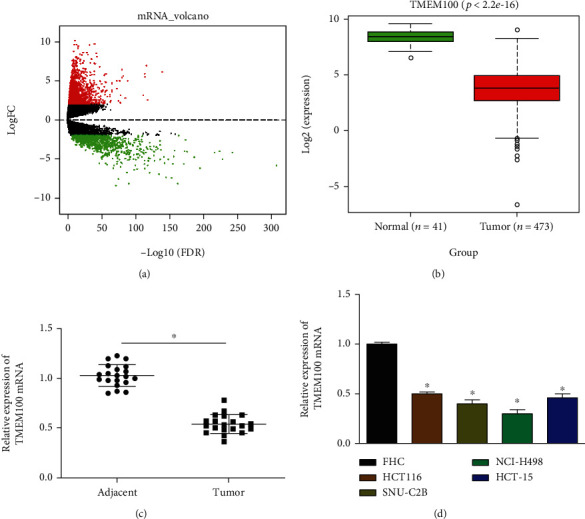
TMEM100 expression is decreased in CRC tissue and cells. (a) Volcano plot of DEmRNAs. Red for highly expressed genes, and green for lowly expressed ones. (b) Relative expression of TMEM100 in the normal group and tumor group. (c) qRT-PCR determined the TMEM100 expression in the tumor and normal samples. (d) TMEM100 expression in normal human colorectal cells and CRC cells. ^∗^*p* < 0.05.

The mRNA expression data of CRC were acquired from the TCGA-COAD dataset. Differentially expressed mRNAs (DEmRNAs) were screened by using “edgeR” package (∣logFC∣>2, *p*adj < 0.05). 2069 DEmRNAs were discovered (Figure 1(a)), among which TMEM100 was significantly poorly expressed in the tumor tissue (Figure 1(b)). Research has unveiled that TMEM100 is able to suppress GC cell migration and enhance the chemosensitivity of GC cells [13]. Additionally, the downregulation of TMEM100 is found to be remarkably linked to the poor prognosis of NSCLC sufferers [14, 15]. Here, we assessed the TMEM100 expression in tumor and adjacent normal tissues via qRT-PCR, discovering that TMEM100 expression was noticeably low in CRC tissue in comparison with the expression in adjacent normal tissue (Figure 1(c)). Besides, TMEM100 expression in normal colorectal cell line FHC and CRC cell lines HCT116, NCI-H498, SNU-C2B, and HCT-15 was detected, finding that compared with normal cell line, TMEM100 was markedly downregulated in CRC cell lines (Figure 1(d)). Taken together, the above results indicated that TMEM100 had low expression in CRC tissue and cells.

### 3.2. TMEM100 Restrains CRC Cell Proliferation

**Figure 2:**
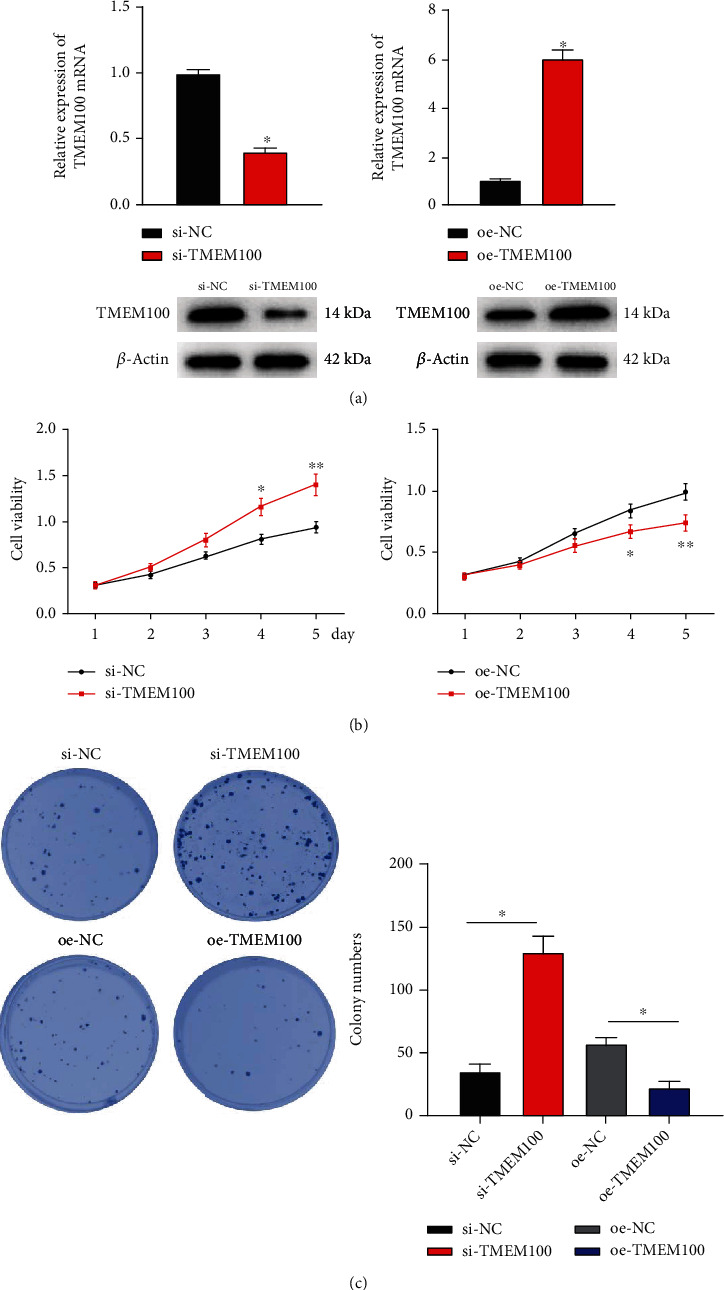
TMEM100 inhibits NCI-H498 cell proliferation. (a) TMEM100 expression upon TMEM100 silencing or overexpression analyzed by qRT-PCR. (b) MTT assay was performed to evaluate NCI-H498 cell proliferative ability upon TMEM100 silencing or overexpression. (c) The colony formation capacity of NCI-H498 cells upon TMEM100 silencing or overexpression was measured by colony formation assay. ^∗^*p* < 0.05, ^∗∗^*p* < 0.01.

To explore the role of TMEM100 in proliferation and colony formation of CRC cells, NCI-H498 cells were treated with TMEM100 siRNA (si-TMEM100) and TMEM100 overexpression vector (oe-TMEM100). qRT-PCR revealed that TMEM100 was markedly knocked down after transfection with si-TMEM100 but significantly upregulated after transfection with oe-TMEM100, which uncovered high transfection efficiency (Figure 2(a)). MTT assay suggested that the NCI-H498 cell proliferation ability was noticeably enhanced upon TMEM100 silencing but markedly weakened upon TMEM100 overexpression (Figure 2(b)). Additionally, the colony formation capacity of NCI-H498 cells was remarkably raised upon TMEM100 knockdown but significantly decreased upon TMEM100 overexpression (Figure 2(c)). Collectively, the above experiments demonstrated that TMEM100 could suppress CRC cell proliferation.

### 3.3. TMEM100 Represses CRC Cell Migration and Invasion

**Figure 3:**
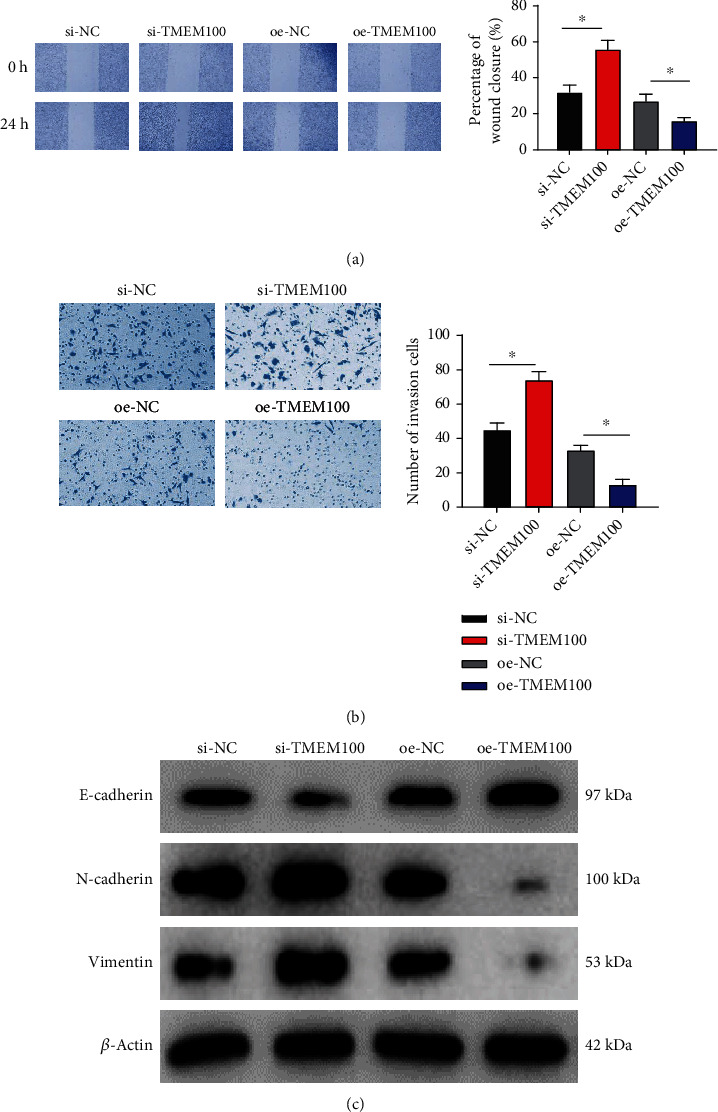
TMEM100 restrains the migration and invasion of NCI-H498 cells. (a) Scratch healing assay detected the migration of NCI-H498 cells upon TMEM100 silencing and overexpression. (b) Transwell assay assessed invasion of NCI-H498 cells upon TMEM100 silencing and overexpression. (c) Western blot tested the effect of TMEM100 on EMT of NCI-H498 cells. ^∗^*p* < 0.05.

To explore the influence of TMEM100 on the migration and invasion of CRC cells, scratch healing and Transwell assays were adopted. The former indicated that the NCI-H498 cell migratory ability was noticeably enhanced upon TMEM100 silencing but markedly inhibited upon TMEM100 overexpression (Figure 3(a)). The latter showed that the NCI-H498 cell invasion capacity was remarkably increased upon TMEM100 knockdown but markedly repressed upon TMEM100 overexpression (Figure 3(b)). Moreover, western blot determined the influence of TMEM100 on epithelial-mesenchymal transition (EMT) of NCI-H498 cells, unveiling that knockdown of TMEM100 silenced the epithelial marker (E-cadherin) of NCI-H498 cells but upregulated the mesenchymal markers (vimentin and N-cadherin). On the contrary, overexpressing TMEM100 elevated E-cadherin but downregulated N-cadherin and vimentin (Figure 3(c)). These results suggested that the high expression of TMEM100 in NCI-H498 cells could inhibit cell migration and invasion.

### 3.4. TMEM100 Represses CRC Cell Growth by Regulating TGF-*β* Signaling Pathway

**Figure 4:**
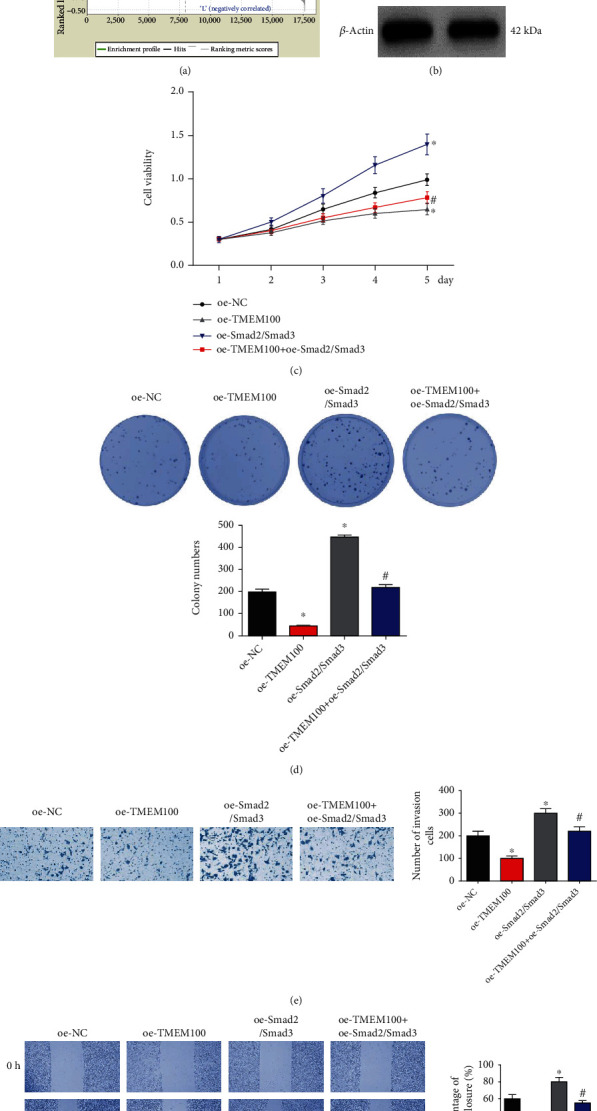
TMEM100 suppresses CRC cell growth by regulating TGF-*β* signaling pathway. (a) The signaling pathway regulated by TMEM100 was analyzed by GSEA. (b) Western blot assessed the expression of TGF-*β* signaling-related proteins upon TMEM100 overexpression. The proliferative, colony formative, invasive, and migratory abilities of NCI-H498 cells were determined by conducting (c) MTT assay, (d) colony formation assay, (e) Transwell assay, and (f) scratch healing assay upon simultaneously overexpressing Smad2/Smad3 and TMEM100. ^∗^Compared with oe-NC, *p* < 0.05; ^#^compared with oe-Smad2/Smad3, *p* < 0.05.

Gene set enrichment analysis (GSEA) pointed out that TMEM100 was markedly concentrated in the TGF-*β* signaling pathway (Figure 4(a)). Therefore, we further investigated whether TMEM100 regulated the TGF-*β* signaling pathway. Notably, western blot indicated that TMEM100 overexpression reduced TGF-*β* expression, as well as the phosphorylation of Smad2 and Smad3 (Figure 4(b)). To verify that TMEM100 modulated malignant growth of CRC cells via the TGF-*β* signaling pathway, we simultaneously overexpressed Smad2/Smad3 and TMEM100, finding that the inhibitory effect of TMEM100 overexpression on the progression of NCI-H498 cells were all reversed by overexpressing Smad2/Smad3 (Figures 4(c)–4(f)). Collectively, it could be concluded that TMEM100 suppressed CRC cell growth by repressing TGF-*β* signaling pathway activation.

## 4. Discussion

CRC is a kind of malignancy worldwide that poses a huge threat to human health [16]. CRC metastasis is a primary cause of death in CRC patients [17]. In the United States, CRC patients with focal hepatic lesion have a 5-year overall survival rate of 90%, while that of patients with distant metastasis is only 5%-8% [1]. Besides, EMT is the reason contributing to the distant metastasis of CRC sufferers [18]. Consequently, it is of great importance to find a new therapeutic method for CRC, especially for metastatic CRC.

TMEM100 is located at 17q32 and encodes a 134-amino acid protein. A recent study has indicated that upregulation of TMEM100 activity *in vivo* inhibited lung metastasis of gastric cancer cells, suggesting that TMEM100 may be linked with tumor invasion and metastasis [13]. In this study, TMEM100 was discovered to be noticeably downregulated in CRC patients via bioinformatics analysis and *in vitro* experiments. But the role of TMEM100 in CRC has not been reported yet. As a result, we further studied the function of TMEM100 in CRC cells, discovering that TMEM100 knockdown could facilitate progression of CRC cells. On the contrary, overexpression of TMEM100 had an inhibiting effect on CRC cell progression. EMT is a major participant in regulating tumor metastasis [19]. Downregulation of E-cadherin and up-regulation of N-cadherin and vimentin are key characteristics of EMT, resulting in unstable adhesion junctions. EMT has been confirmed to be associated with aggressive or metastatic phenotypes in CRC. In our study, knockdown of TMEM100 promoted EMT in CRC, while overexpression of TMEM100 caused an opposite result. These results suggested that TMEM100 inhibited the EMT process by affecting the expression of CRC phenotypic proteins, thus increasing invasive and migratory abilities of CRC cells.

To have a deeper understanding into the mechanism of TMEM100 in CRC, GSEA was used to find that TMEM100 was mainly enriched in the TGF-*β* signaling pathway. The signaling pathway is a series of transforming growth factor-mediated signal transduction processes, which is important in growth, development, and differentiation of cells and tissues, as well as in the occurrence, development, and metastasis of a number of tumors [20]. Studies have unveiled that TGF-*β* suppresses tumor cell growth at the early stage of tumorigenesis but fosters tumor progression at the advanced stage [21–23]. In this study, we discovered that TMEM100 overexpression could inhibit phosphorylation of Smad2 and Smad3, as well as the expression of TGF-*β*1 in CRC cells, but the inhibitory effect of TMEM100 overexpression on the progression of CRC cells could be reversed by overexpressing Smad2/Smad3. Cai et al. [24] reported that tumor-associated macrophages could promote the EMT process via the TGF-*β*/Smad2, 3-4/Snail signaling pathway. Additionally, epidermal growth factor (EGF) can induce EMT of breast cancer cells via the phospho-Smad2/3 Snail signaling pathway [25]. These evidences strongly demonstrated that by restraining the activation of the TGF-*β* signaling pathway with TMEM100, Smad2/3 phosphorylation, and phenotypic transformation of EMT process in CRC cells can be inhibited, and finally, the malignant progression of CRC can be suppressed.

**Figure 5:**
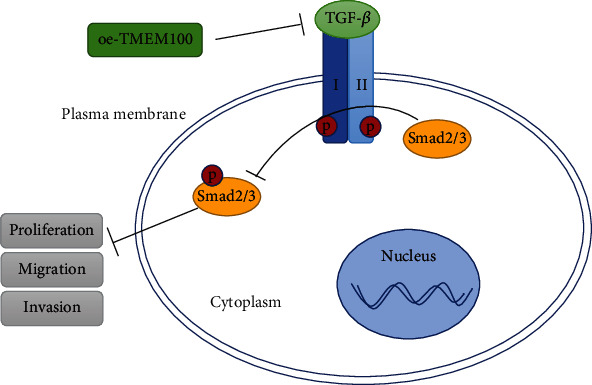
Mechanism diagram of TMEM100 inhibiting CRC. TMEM100 inhibits TGF-*β* activation by suppressing the interaction between TGF-*β* ligand and its homology type I and type II one-way transmembrane receptors, thereby inhibiting its Smad2/Smad3 phosphorylation. Ultimately, progression of CRC cells was hampered. Red circles represent phosphorylation. I and II represent TGF-*β* type I and type II receptors, respectively.

All in all, the present research uncovered the molecular mechanism of TMEM100 in CRC (Figure 5). As a tumor suppressor gene, TMEM100 restrained CRC malignant progression by regulating the TGF-*β* signaling pathway. It will provide new ideas and theoretical basis for developing treatment strategies for colorectal cancer and other malignant diseases.

However, there are still some deficiencies in our research. For example, only the role of TMEM100 in CRC cell lines was studied, and no corresponding *in vivo* experiments were conducted. We will conduct *in vivo* experiments and clinicopathological correlation analysis in subsequent studies to obtain more accurate and reliable results.

## Data Availability

The data used to support the findings of this study are included within the article. The data and materials in the current study are available from the corresponding author on reasonable request.

## Supplementary Materials

Supplementary Materials Supplementary Table 1: primer sequences in qRT-PCR. Supplementary Table 2: antibodies for western blot.
