# Supplementary material for: TMEM100 Modulates TGF-β Signaling Pathway to Inhibit Colorectal Cancer Progression
Source: Gastroenterol Res Pract. 2021 Aug 11;2021:5552324. doi: 10.1155/2021/5552324 (PMC8373494; doi:10.1155/2021/5552324)
Supplement: Supplementary Materials — Supplementary Table 1: primer sequences in qRT-PCR. Supplementary Table 2: antibodies for western blot. [file 5552324.f1.docx]

**Table**

**Supplementary Table 1 Primer sequences in qRT-PCR**

| Gene | Sequences |
| --- | --- |
| TMEM100 | F: 5'-CCGGAATTCCGGATGACTGAAGAGCCCAT-3' |
|  | R: 5'-CGGGATCCCGTCAAGCAAACAAGCTT -3' |
| GAPDH | F: 5'- GGAGCGAGATCCCTCCAAAAT-3' |
|  | R: 5'- GGCTGTTGTCATACTTCTCATGG-3' |

**Supplementary** **Table 2 Antibodies for Western blot**

| Antibody | Western blot | Specificity | Company |
| --- | --- | --- | --- |
| N-cadherin | 1 µg/ml | Rabbit polyclonal | Abcam, China |
| E-cadherin | 1/10000 (1/500) | Rabbit monoclonal | Abcam, China |
| TMEM100 | 5 µg/mL | Rabbit monoclonal | Sigma, USA |
| Vimentin | 1/1000 | Rabbit monoclonal | Abcam, China |
| TGF-β1 | 0.5-4 µg/ml. | Rabbit polyclonal | Abcam, China |
| p-Smad2 | 1/500 | Rabbit polyclonal | Abcam, China |
| Smad2 | 1/1000 | Rabbit monoclonal | Abcam, China |
| p-Smad3 | 1/500 | Rabbit polyclonal | Abcam, China |
| Smad3 | 1/1000 | Rabbit monoclonal | Abcam, China |
| IgG H&L (HRP) | 1:3000 | Rabbit monoclonal | Abcam, China |
| β-actin | 1/5000 | Rabbit monoclonal | Abcam, China |
